# A comparison of two cellular delivery mechanisms for small interfering RNA

**DOI:** 10.14814/phy2.12286

**Published:** 2015-02-03

**Authors:** Virginijus Valiunas, Hong-Zhang Wang, Ling Li, Chris Gordon, Laima Valiuniene, Ira S Cohen, Peter R Brink

**Affiliations:** Department of Physiology and Biophysics, Stony Brook UniversityStony Brook, New York

**Keywords:** Endocytosis, gap junction, siRNA delivery

## Abstract

Cellular delivery of small interfering RNAs to target cells of a tissue has the potential to travel by two intercellular pathways. For intimately apposed cells gap junctions allow transport exclusive of the extracellular space. For cells not in intimate contact, exocytotic release of vesicular contents and subsequent retrieval via endocytosis of exosomes and other vesicular contents represent an alternative intercellular delivery system that utilizes the extracellular space. Previous studies have shown siRNA/miRNA transfer from a delivery cell to a target cell via gap junction channels. We hypothesized that siRNA can be delivered via gap junctions and downregulate the expression of a reporter gene, the cyclic nucleotide-gated cation channel gene (mHCN2), in the recipient cells of cell pairs. Whole-cell patch clamp was used to measure the mHCN2-induced current and junctional conductance. The target cells were HEK293 cells that endogenously express Cx43 or HeLaCx43 cells, both transfected with mHCN2. The source cells were HEK293 or HeLaCx43 cells transfected with fluorescent-labeled siRNA targeting mHCN2. We found that siRNA targeting mHCN2 resulted in significant downregulation of mHCN2 currents both in single cells and the recipient cell of a cell pair. In addition we also documented downregulation in target cells that were not in contact with source cells suggesting an extracellular-mediated delivery. To test further for extracellular delivery HEK293/HCN2 or HeLaCx43/HCN2 cells were cultured in medium collected from HEK293 or HeLaCx43 cells transfected with fluorescent-labeled siRNA or fluorescent-labeled morpholino designed to target HCN2. After 24 h single HEK293/HCN2 or HeLaCx43cells showed accumulation of siRNA. The mHCN2 currents were also down regulated in cells with siRNA uptake. Application of 200 nmol/L Bafilomycin A1, which has been shown to affect endosome acidification and endocytotic activity, resulted in a smaller accumulation of fluorescent-labeled siRNA in single target cells. In distinction to siRNA, morpholinos targeting HCN2 exhibited greatly reduced extracellularly mediated transfer while in cell pairs, target cells exhibited reduced HCN2 currents consistent with effective gap junction-mediated delivery.

## Introduction

Cellular delivery of microRNAs (micRNA) and small interfering RNA (siRNA) might be accomplished by multiple intercellular pathways. One form is cell-to-cell delivery mediated by gap junction channels where solutes move directly from one cytoplasm to another intercellularly (Valiunas et al. 2005; Brink et al. 2012; Mittelbrunn and Sanchez-Madrid 2012). One common alternative for cell-to-cell delivery is extracellularly derived transport as a common final pathway to a target cell (Brink et al. 2012). Although other pathways for cell-to-cell transfer have been reported (nanotubes, RNA receptors (Rustom et al. 2004; Cohen and Xiong 2011)) extracellular and gap junction-based delivery have demonstrated the ability to transfer siRNA from one cell to another. Since therapeutic use of siRNA requires delivery to a target cell, our purpose is to define the efficacy of each pathway in order to optimize cell-based siRNA delivery.

The formation of functional gap junction channels between closely apposed cells creates an effective cytoplasmic continuum for a variety of small molecules (Valiunas et al. 2002; Goldberg et al. 2004; Kanaporis et al. 2008, 2011; Brink et al. 2012). The movement of small molecules between cells coupled by gap junctions is governed by channel open probability and selectivity. Open probability has been determined to be between 0.5 and unity (Brink et al. 1996; Christ and Brink 1999) while selectivity is dependent on solute size and charge (Valiunas et al. 2002; Kanaporis et al. 2008, 2011) and the type of connexin or connexins comprising the gap junction channels (Harris 2001; Ayad et al. 2006). Interestingly, siRNA analogs (morpholinos) with minor diameters of ∼1.0 nm have been shown to be permeable to gap junction channels composed of connexin43 (Cx43), (Valiunas et al. 2005). The third factor essential for rapid cell-to-cell transit of solutes is the number of functional channels between any two adjacent cells. For example, cAMP flux is linearly proportional to Cx43 macroscopic junctional conductance (Kanaporis et al. 2008).

Exocytosis/endocytosis-(Cohen and Xiong 2011; van den Boorn et al. 2011; Bang and Thum 2012; Mittelbrunn and Sanchez-Madrid 2012) based delivery includes diffusion of expelled vesicular contents, including exosomes, via the extracellular space. This system is able to transfer larger solutes with little or no dependence on solute size and charge to the limit of vesicular diameter. Exocytosis, endocytosis, and exosomes are common features of most if not all mammalian cells. But there are important rate determining steps for exocytotic/endocytotic delivery from one cell to another. First is the rate at which vesicles are released or absorbed/cell/time unit, second is the number of deliverable molecules or particles within any particular vesicle, third is the fate of any endocytosed vesicle in the target cell, and fourth the concentration dilution imposed by the semiinfinite or infinite in vivo extracellular volumes (Brink et al. 2012). Only one in vitro study has attempted to define vesicle-based delivery relative to gap junction-mediated delivery. Lim et al. (2011) provided evidence for cell-to-cell transfer of an siRNA where the results suggested that the majority of siRNA was being delivered to target cells via gap junctions with a smaller component of silencing resulting from exocytosis/endocytosis.

To address the utility of the gap junction and extracellular-mediated pathways (exocytotic/endocytotic) we first demonstrate that a fluorescently tagged siRNA targeting a reporter gene, HCN2 is able to reduce the number of functioning channels in single whole-cell patch clamp. We also demonstrate the same for a morpholino targeting HCN2. Second, we load either HEK or HeLa cells that did not express HCN2 with either tagged siRNA or morpholino for 24 h yielding an intracellular concentration of ∼300 nmol/L. We cocultured the loaded cells for an additional 24 and 48 h with target HEK or HeLa cells expressing HCN2. Extracellular volumes were 1 mL. Fluorescently tagged morpholinos targeting HCN2 did not traffic efficiently from cell to cell via the extracellular space. This same morpholino was able to transfer from cell to cell via gap junctions and effectively reduce the HCN2-induced current in cell pairs comprised of a source cell and a target cell. The siRNA targeting HNC2 was able to effectively reduce HCN2 currents in target cells via the extracellular delivery pathway and the gap junction-mediated delivery.

Our results demonstrate both extracellular-mediated delivery and gap junction-mediated delivery are possible. Pharmacological inhibition of vacuolar proton ATPase in endosomes by Bafilomycin A1 inhibited knockdown of HCN2 currents relative to controls suggesting that endocytotic entrance of siRNA into a target cell is pH dependent (Wang et al. 2014; Matsuda et al. 2014). Junctional conductance was also reduced by 30% with the same treatment. These results also suggest that the siRNA/morpholino sequence is in part responsible for determining whether extracellular delivery or gap junction-mediated cellular delivery is utilized.

## Materials and Methods

### Cells and culture conditions

Experiments were carried out on HeLa cells stably transfected with mCx43 or HEK293 cells that were endogenously expressing Cx43. HeLa and HEK293 cells were grown in DMEM (Gibco BRL, Life Technologies, Grand Island, NY), supplemented with 10% FCS (Hyclone), 100 μg/mL streptomycin (Gibco BRL, Life Technologies), and 100 U/mL penicillin (Gibco BRL, Life Technologies). The medium also contained 100 μg/mL hygromycin (Sigma, St.Louis, MO) or 0.5 μg/mL geneticin (Gibco Invitrogen, Life Technologies, Carlsbad, CA).

The cells were passaged weekly, diluted 1:10, and kept at 37°C in a CO_2_ incubator (5% CO_2_/95% ambient air). Culture conditions for these cells have been previously published in complete detail (Valiunas et al. 2000, 2001; Gemel et al. 2004; Valiunas et al. 2009). Electrophysiological measurements and siRNA transfer experiments were carried out on cells cultured for 1–3 days. HeLa stably transfected with Cx43 and the endogenously expressing HEK293 were chosen because both robustly express Cx43.

### Cell preparation for siRNA and morpholino transfer studies

siRNA transfer through gap junction channels and via extracellular delivery was investigated using single HeLaCx43 and HEK293 cells. For the detection of siRNA transfer, the cells were transfected with mHCN2, a hyperpolarization-activated, cyclic nucleotide-gated Na^+^/K^+^ channel. A full-length mHCN2 cDNA was subcloned into eukaryotic pIRES2-eGFP vector (Clontech Laboratories, Inc., Mountain View, CA) HeLaCx43 or HEK293 cells were transfected with the cDNA using Lipofectamine 2000 (Invitrogen) following the manufacturer's instructions. Production, characterization, culture conditions, staining, and visualization of these cells have been described previously in Valiunas et al. (2009).

A 21 nucleotide long Cy-3 labeled predesigned siRNA against mHCN2 was obtained from Ambion/Applied Biosystems (Life Technologies). The template sequences used in this study: CGGCUCAUCCGAUAUAUCCtt (sense) and GGAUAUAUCGGAUGAGCCGtg (antisense). HeLaCx43 and HEK293 cells were transfected with siRNA targeting mHCN2 using Lipofectamine RNAiMAX (Invitrogen) as directed with the manufacturer's protocols.

Morpholino 25-mer antisense oligo with a 3′-lissamine red emitting fluorescent tag targeting HCN2 was made by Gene Tools. The sequence of oligonucleotides: TTGGTCCTCTCCCTGCCCCTCACCT. Endo-Porter was used according to instructions from manufacturer's protocol to deliver morpholino oligos into the cells.

Source cells were transfected with Cy-3 siRNA or morpholino 24 h before they were cocultured with mHCN2/eGFP-transfected cells. Heterologous pairs of Cy-3 siRNA (or morpholino) transfected cell (red fluorescence) and mHCN2/eGFP-transfected cell (green fluorescence) were chosen to study gap junction-mediated siRNA (morpholino) delivery. Single mHCN2/eGFP-transfected cells not in direct contact with source cells (Cy-3 siRNA or morpholino transfectants) were studied for extracellularly derived delivery. For endocytic transport inhibition (Bayer et al. 1998) the cells were incubated with 200 nmol/L of Bafilomycin A1 (Sigma).

#### Conditioned medium experiments

HeLaCx43 and HEK293 cell were transfected with Cy3siRNA and incubated for 24 h. After 24 h the cells were repeatedly washed with PBS and fresh medium and left in culture for another 24 h. Then, the medium from the last rinse was collected, filtered to remove cells, and perfused onto another population of cells expressing mHCN2/eGFP. Fluorescent imaging and electrophysiological measurements were performed on these cells after 24–48 h of incubation with collected conditioned medium.

### Electrophysiological measurements

Experiments were carried out on single cells and cell pairs using the whole-cell/perforated patch voltage-clamp technique to control the membrane potential and to measure membrane currents of the cell as previously described (Valiunas et al. 2005, 2009). For electrical recordings, glass coverslips with adherent cells were transferred to an experimental chamber mounted on the stage of an inverted microscope (Olympus- IX71). During experiments, the cells were superfused with bath solution at room temperature (∼22°C) containing (mmol/L): NaCl 137.7; KCl 5.4; MgCl_2_ 1; CaCl_2_ 2; HEPES 5 (pH 7.4); glucose 10; 2 mmol/L CsCl, BaCl_2_, and CdCl_2_ were also added. The patch pipettes were filled with solution containing (mmol/L): K^+^ aspartate^−^, 120; NaCl, 10; MgATP, 3; HEPES, 5 (pH 7.2); EGTA, 10 (pCa ∼8); filtered through 0.22 μm pores. Patch pipettes were pulled from glass capillaries (code GC150F; Harvard Apparatus) with a horizontal puller (DMZ-Universal; Zeitz-Instrumente, Martinsried, Germany). When filled, the resistance of the pipettes measured 1–4 MΩ.

To demonstrate direct cell-to-cell transfer of siRNA from one cell of a pair to another, one patch electrode containing 0.4 mmol/L of tagged siRNA was used to obtain whole-cell patch mode and fluorescence was then monitored. At a later time point a second electrode was positioned onto the recipient cell of the pair and whole-cell configuration was obtained to determine junctional conductance (Valiunas et al. 2002). Monitoring of HCN2 currents was done in whole-cell patch clamp using a previously described step protocol (Valiunas et al. 2009).

### Signal recording, analysis, and statistics

Voltage and current signals were recorded using patch-clamp amplifiers (Axopatch 200, Molecular Devices, Sunnyvale, CA). The current signals were digitized with a 16 bit A/D-converter (Digidata 1322A; Molecular Devices) and stored within a personal computer. Data acquisition and analysis were performed with pClamp9 software (Molecular Devices). Statistical analysis was performed using SigmaStat (Jandel Scientific, San Jose, CA). The Mann–Whitney Rank Sum test was used for all cases unless otherwise noted. A value of *P* < 0.05 was assumed as significantly different. The results are presented as means ± SEM.

## Results

### Tagged siRNA targeting HCN2 can effectively reduce HCN2-induced currents

**Figure 1:**
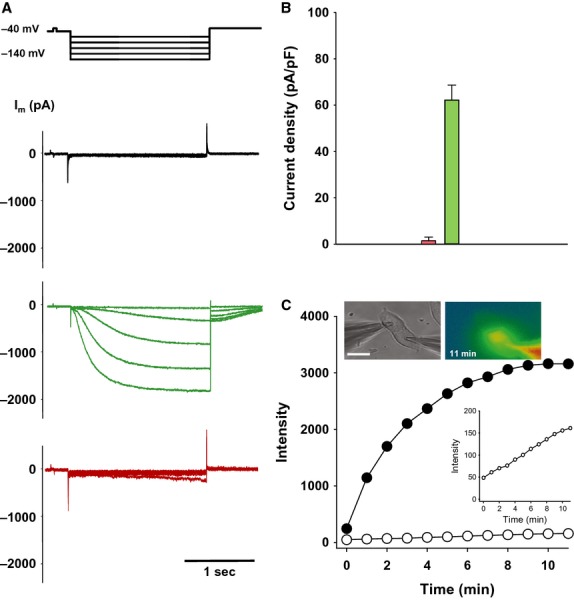
(A–B) Direct transfection of siRNA Cy-3 targeting mHCN2 in single cells. (A) Properties of mHCN2 channels. Voltage protocol (upper panel) and whole-cell currents (*I*_m_) recorded in untransfected (black current traces) and mHCN2-transfected (green current trace) HEK293 cells. Red current traces represent mHCN2-transfected cells that are also transfected with siRNA (siRNA Cy-3) against mHCN2. (B) Average current densities measured at −140 mV from HEK293/HCN2 control cells (green bar: 62.1± 6.7 pA/pF, *n* = 28) and siRNA-transfected cells (pink bar: 1.5 ± 0.5 pA/pF, *n* = 10), *P*<0.001. (C) Detection of cell-to-cell transfer of siRNA in isolated cell pairs. Fluorescent intensity over time for source cell (•) and recipient cell (○). Upper insert: bright field image (left) and fluorescent image in pseudo-colors (right) of a HeLaCx43 cell pair where a patch electrode containing siRNA Cy-3 is in whole-cell configuration with one of the two cells (source cell). The fluorescent image was taken 11 min after establishing a whole-cell patch in the source cell. Fluorescence can be seen in the recipient cell. To enhance visibility of siRNA Cy-3 in the recipient cell, fluorescence is shown here in pseudo-color scale from the lowest intensity (blue, background) to red (highest intensity). After 11 min a second electrode was used to patch the recipient cell and junctional conductance of *g*_j_ = 35 nS was determined). Scale bar, 20 μm Lower insert: expanded scale for recipient cell.

Figure1A and B illustrate the effectiveness of an siRNA that targets HCN2 in a single cell. HEK293 cells were transfected with mouse HNC2 and using the voltage step protocol shown in Fig.1A (upper panel) HCN2-induced currents were readily observable. Transfection for 24 h of the same cells with siRNA targeting HCN2 resulted in a significant reduction in HCN2 currents (Fig.1A lower panel). Figure1B summarizes HCN2 currents measured in HEK293 control cells and cells transfected with siRNA against HCN2 at *V*_*m*_ = −140 mV. siRNA-transfected cells exhibited ∼40 times smaller HCN2 currents versus control cells expressing HCN2 (see Fig.1B for details).

To determine if the siRNA could pass through gap junctions composed of Cx43 one patch electrode used to assess junctional conductance was loaded with Cy-3-labeled siRNA. Whole-cell patch was attained using the Cy-3-containing electrode and fluorescence monitored over time. The upper insert in Fig.1C shows an example of transfer of the tagged 21-mer siRNA delivered via electrode to a source cell of HeLaCx43 cell pair. Within 11 min transfer to the adjacent cell was observed. Figure1C shows fluorescence intensity determined at different time intervals in both the source (•) and recipient cell (○). Subsequently the whole-cell configuration was obtained at the end of tagged siRNA transfer to the recipient cell and a junctional conductance of 35nS was determined by measuring recorded transjunctional current (not shown). This example shows that when siRNA is delivered via an electrode, an intercellular transit occurs to a coupled target cell similar to morpholino transfer (Valiunas et al. 2005).

**Figure 2:**
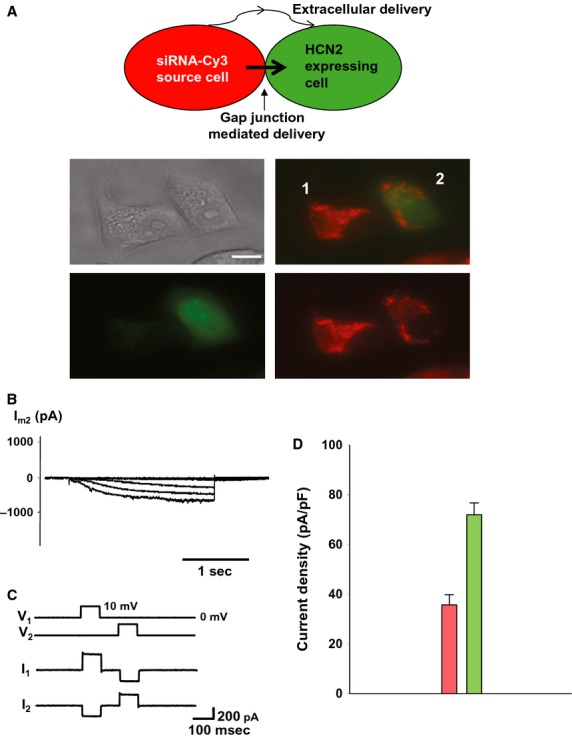
Cell-to-cell transfer of siRNA. (A) Schematic in the upper panel illustrates experimental conditions. Middle panel: cell 1- HeLaCx43 cell transfected with siRNA Cy-3 (red), cell 2- recipient cell transfected with mHCN2 gene and eGFP (green). After 24 h in coculture red-labeled siRNA Cy-3 can be detected in the recipient cell (green) of the pair (lower panel). Scale bar, 10 μm. (B) *I*_m_ elicited in cell 2 (green) showed inhibited HCN2 currents. (C) Voltage steps (10 mV, 50 ms) applied to cell 1- and cell 2-induced currents in the cell pair above showing gap junction coupling of *g*_j_ = 22 nS. (D) Averages of current densities measured from single control cells (green bar: 71.9 ± 4.7 pA/pF, *n* = 54) and cells from the pair with siRNA-transfected cells (pink bar: 35.7 ± 4.1 pA/pF, *n* = 51), *P* < 0.001.

Twenty-four hour coculture of cell pairs composed of one source cell (HeLaCx43) transfected with tagged siRNA and the other cell (HEK293 or HeLaCx43) expressing eGFP and HCN2 (Fig.2A) demonstrate siRNA Cy-3 transfer from a source cell and subsequently reduced HCN2-induced current in the recipient cell (Fig.2B). Previous to coculturing, one population of HeLaCx43 cells was transfected with Cy-3 siRNA for 24 h. The cell pair shown in Fig.2A was dual whole-cell patch clamped and shown to have a junctional conductance of 22 nS (Fig.2C). HCN2 current densities measured from recipient cells of a pair with visible Cy-3 content (like cell 2 in Fig.2A) were summarized in Fig.2D. On average, such cells exhibited ∼50% of the HCN2-induced current compared to control HCN2-expressing cells (35.7 ± 4.1 pA/pF vs. 71.9 ± 4.7 pA/pF, Fig.2D). These data are consistent with a gap junction-mediated delivery but do not exclude an extracellular delivery pathway.

### Evidence of extracellularly mediated delivery of siRNA

**Figure 3:**
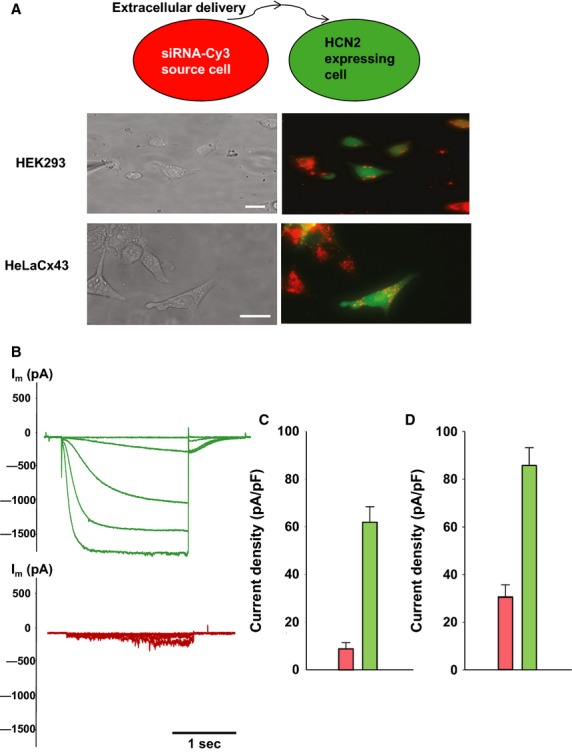
Extracellular delivery of siRNA. (A) Upper panel: schematic of experimental conditions for HEK293 cells. Middle panel: coculture of HEK293 cells expressing mHCN2 and eGFP with siRNA Cy-3 preloaded HEK293 cells for 48 h. eGFP cells not in contact with the siRNA Cy-3 cells contain siRNA Cy-3 with vesicular compartments. Scale bar, 10 μm. Lower panel: coculture of HeLaCx43 cells expressing mHCN2 and eGFP with siRNA Cy-3 preloaded HeLaCx43 cells for 24 h. eGFP cells not in contact with the siRNA Cy-3 cells contain siRNA Cy-3 with vesicular compartments. Scale bar, 20 μm. (B) Currents from single HEK293 mHCN2-expressing cells (green traces) and HCN2 currents from those single cells containing siRNA Cy-3 vesicular compartments (red traces). (C) Histogram for all HEK293 cells data collected: control, HCN2-expressing cells (green bar: 62.1 ± 6.5 pA/pF, *n* = 28) and HCN2-expressing cells containing siRNA Cy-3 vesicular compartments (pink bar: 9.1 ± 2.5pA/pF, *n* = 25), *P* < 0.001. (D) Average of current densities measured from single control HeLaCx43/HCN2 cells (green bar: 86.0 ± 7.4 pA/pF, *n* = 18) and from single HeLaCx43/HCN2 cells containing siRNA Cy-3 vesicular compartments (pink bar: 30.7 ± 5.3 pA/pF, *n* = 16), *P* < 0.001.

Within the same cocultures recipient cells containing Cy-3 siRNA were found that had no apparent gap junction-mediated connection. Figure3A (middle panel) shows examples of green HCN2/eGFP expressing recipient HEK293 cells that contain Cy-3 siRNA (red) but that are not in contact with source cells (HEK293 transfected with Cy-3 siRNA, red cells). The lower panel in Fig.3A shows a similar example with HeLaCx43 cells. In Fig.3B typical HCN2-induced currents for HEK293 control recipient cells (no siRNA) and Cy-3 siRNA-containing recipient cells are illustrated. Cy-3 siRNA-containing cells possessed significantly reduced HCN2-induced currents. The data are summarized in Fig.3C. The cells containing a significant (easily visible) amount of siRNA exhibited ∼15% of the HCN2 currents observed in control cells. The summary of currents recorded from HeLaCx43 control cells (HCN2/eGFP expressing) and cells containing Cy-3 siRNA vesicular compartments are shown in Fig.3D. HCN2-induced currents recorded from HeLaCx43 cells also containing siRNA Cy-3 were reduced to approximately 1/3 those recorded from control HeLaCx43 cells. Nonexposed HeLa cells usually exhibited larger HCN2-induced currents than HEK293 cells.

**Figure 4:**
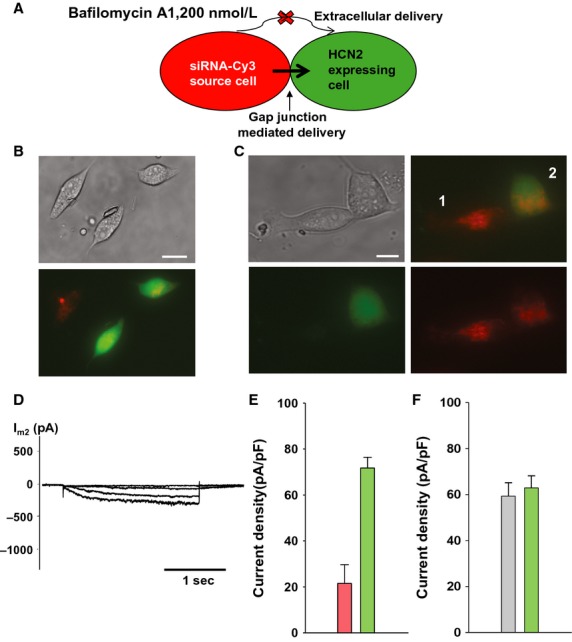
Gap junction-mediated transfer of siRNA in the presence of Bafilomycin. (A) Upper panel: schematic of experimental conditions. (B) In the presence of Bafilomyocin A1 (24 h) no significant siRNA Cy-3 transfer is seen in HeLaCx43 cells. Scale bar, 20 μm. (C) Middle and lower panels: HeLaCx43 cell pair incubated with Bafilomyocin A1 for 24 h. In this case siRNA Cy-3 is observed in the recipient (eGFP) cell. Scale bar, 10 μmol/L. (D) *I*_m_ elicited in cell 2 (green cell) showed inhibited HCN2 currents. (E) Averages of current densities measured from single control cells (green bar: 71.9 ± 4.7 pA/pF, *n* = 54) and cells from the pair with siRNA-transfected cells (pink bar: 21.8 ± 8.2 pA/pF, *n* = 9) incubated with Bafilomycin A, *P* < 0.001. (F) Summary of HCN2 currents recorded from HEK/HCN2 cells incubated with Bafilomycin A1 for 24 h (gray bar: 59.5 ± 5.8 pA/pF, *n* = 66 and control cells (green bar: 63.1 ± 5.2 pA/pF, *n* = 62), *P* = 0.474.

To further investigate the extracellular delivery pathway we performed experiments as shown in Fig.3 but in the presence of the vacuolar proton ATPase inhibitor Bafilomycin A1 (Bayer et al. 1998) with HeLaCx43 cells (Fig.4). Figure4B shows the effect of Bafilomycin Al on extracellularly mediated transfer. HeLaCx43 cells expressing HCN2 were cocultured with HeLaCx43 transfected with siRNA Cy-3 in the presence of 200 nmol/L Bafilomycin A1. Bafilomycin A1 greatly reduced but did not eliminate extracellularly (exocytotic/endocytotic) mediated transport. Quantification of the effect of Bafilomycin A1 transfer is shown in Fig. 6.

An important demonstration of gap junction-mediated delivery is shown in Fig.4C–F. Figure4C shows a heterologous pair of siRNA- and HCN2-transfected HeLaCx43 cells cocultured in the presence of 200 nmol/L of Bafilomycin A1. siRNA transfer detected in the HCN2-expressing cell is consistent with gap junction-mediated delivery. Figure4D shows HCN2-induced current recorded from the siRNA recipient cell2. The average such current recorded from nine preparations was significantly smaller (21.8 ± 8.2 pA/pF) in comparison with control cells (71.9 ± 4.7 pA/pF) (Fig.4E).

Another series of experiments were performed to test whether Bafilomycin A1 affects HCN2 currents. Summary data of these experiments are illustrated in Fig.4F which shows the averages of HCN2-induced currents recorded from HEK293/HCN2 cells incubated with Bafilomycin A1 and those from control HEK293/HCN2 cells. There was no statistical difference between these two groups (59.5 ± 5.8 pA/pF vs. 63.1 ± 5.2 pA/pF, *P* = 0.474) showing that Bafilomycin A1 had no significant effect on HCN2-induced current amplitude.

The effects of Bafilomycin A1 on junctional conductance were also determined. After 24 h of exposure to 200 nmol/L Bafilomycin A1 junctional conductance was reduced 22% from a control value of 25.4 ± 2.6 nS (*n* = 12) to 19.9 ± 2.9 (*n* = 11) in HeLa cells. In HEK293 cells the gap junction conductance was reduced by ∼54% from a control value of 15.6 ± 2.1 nS (*n* = 13) to 7.2 ± 1.6 nS (*n* = 12).

#### Conditioned medium experiments

**Figure 5:**
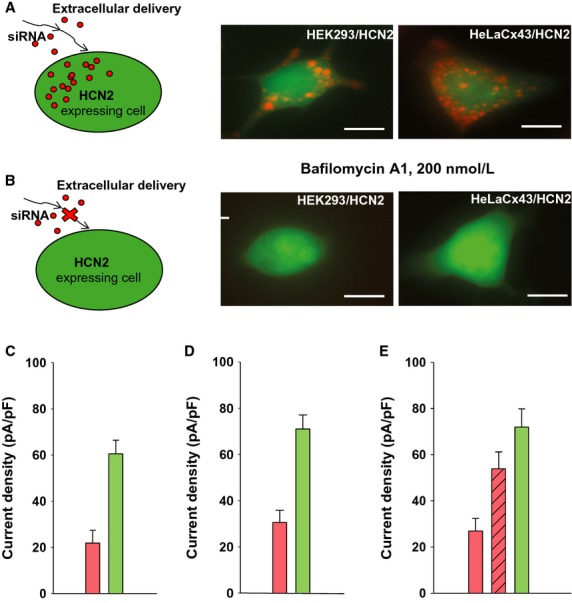
“Conditioned medium” approach. HeLaCx43 and HEK293 cells were transfected with siRNA Cy-3 for 24 h. The medium was repeatedly washed at the 24 h mark and the cells were left in culture for another 24 h. Then the medium from the last rinse was collected, filtered to remove cells, and was perfused onto another population of cells that expressed eGFP and HCN2 and cultured for 24 h. (A) Experimental conditions (left panel) and HEK293/HCN2 (middle panel) and HeLaCx43/HCN2 cells after 24 h in conditioned medium. Both type of cells show vesicular accumulation of siRNA Cy-3 with a bright red fluorescence staining in perinuclear region. (B) HEK293/HCN2 and HeLaCx43/HCN2 cells cultured for 24 h in conditioned medium in the presence of Bafilomycin A1 (left panel: experimental conditions). No visible siRNA transfer was detected. Scale bars 10 μm. (C–D) Summary of HCN2 current data collected from single cells in conditioned medium. (C) HEK/HCN2 cells: pink bar-cells containing siRNA Cy-3 vesicular compartments (21.9 ± 5.6 pA/pF, *n* = 20); green bar-control cells (60.5 ± 5.9 pA/pF, *n* = 25), *P* < 0.001. (D) HeLaCx43/HCN2 cells: pink bar-cells from conditioned medium (30.6 ± 5.2 pA/pF, *n* = 21); green bar: control cells (71.0 ± 6.1 pA/pF, *n* = 27), *P* < 0.001. (E) HeLaCx43/HCN2 cells in conditioned medium treated with Bafilomycin A1 exhibited significant larger currents (striped pink bar: 53.9 ± 7.3 pA/pF, *n* = 18) as compared to cells with no Bafilomycin1 treatment (pink bar: 26.9 ± 5.5 pA/pF, *n* = 15), *P* = 0.002; control cells: green bar (71.9 ± 7.9 pA/pF, *n* = 19). t-test: *P* = 0.102 and *P* < 0.001, control versus Bafilomycin-treated and not-treated cells, respectively.

To better define the extracellular delivery pathway delivery, cells were first transfected with Cy-3 siRNA for 24 h. The cells were then washed repeatedly and then incubated for another 24 h in fresh media. The conditioned media, was then collected and used to incubate HEK293 or HeLaCx43 cells expressing HCN2 for 24 h. The conditioned media was replaced with fresh media and images collected which revealed Cy-3 siRNA in cells (Fig.5A). Repeating the same experiment with Bafilomycin A1 mixed into the conditioned media resulted in little or no Cy-3 siRNA in either HEK or HeLa cells (Fig.5B). Single cells were used to record HCN2 currents in conditioned media. Figure5C and D show the summary data collected from single cells using the experimental paradigm of Fig.5A. HCN2 currents were reduced to ∼36% of control in HEK293 cells (Fig.5C) and to ∼43% of control in HeLaCx43 cells (Fig.5D) which contained Cy-3 siRNA vesicular compartments (Fig. 5A) in comparison to control cells. These data (Fig.5A, C, and D) demonstrate that Cy-3 siRNA was delivered via an extracellular route. To confirm such a pathway another series of experiments were performed where extracellular delivery was inhibited by Bafilomycin Al (Fig.5B). Figure5E summarizes data from such experiments showing significant ∼37% reduction (see Fig.5 legend for details) in HCN2 current in HeLaCx43/HCN2 cells incubated in conditioned medium in comparison to control cells. However, in the presence of 200 nmol/L of Bafilomycin A1 in conditioned medium HCN2 currents were not significantly reduced relative to control cells (*t*-test, *P* = 0.102, see Fig.5 legend for details) thus confirming that Bafilomycin A1 effectively inhibits extracellularly mediated delivery.

**Figure 6:**
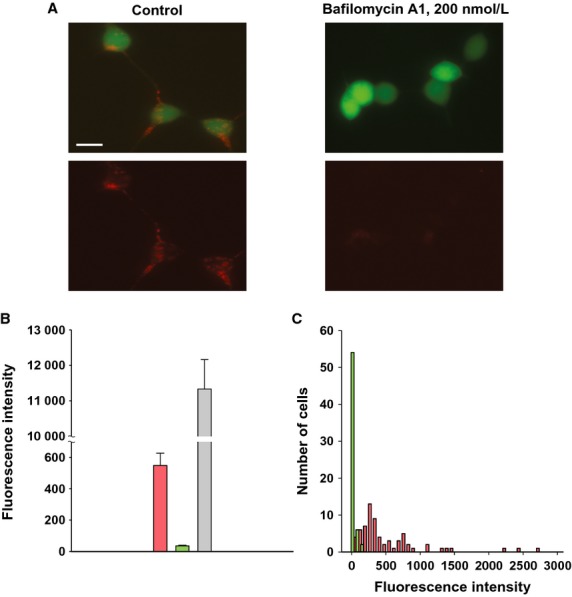
Extracellular transfer efficiency. (A) HEK293/HCN2 cells incubated in conditioned medium for 24 h. Left panel: in control cells typical vesicular accumulation of siRNA Cy-3 resulted in a bright red fluorescence staining in perinuclear region (bottom). In the presence of 200 nmol/L Bafilomycin A1 (right panel) very weak siRNA Cy-3 accumulation was observed. (B) Average of siRNA Cy-3 fluorescence intensity measured from control HEK293/HCN2 cells in conditioned medium (pink bar, 549.1±78.3, *n* = 69), and cells incubated with Bafilomycin A1 (green bar, 35.8 ± 4.3, *n* = 62), *P* < 0.001. For comparison gray bar represents fluorescence intensity average from siRNA-transfected cells (11133.2 ± 831.1, *n* = 12). (C) Histograms of siRNA Cy-3 fluorescence intensity gained from cells incubated in conditioned medium without bafilomycin (pink histogram) and in presence of 200 nmol/L Bafilomycin A1 (green histogram). The number of observations (cells) was plotted versus red fluorescence intensity.

In another series of experiments an attempt was made to evaluate the efficiency of extracellular delivery in HEK293/HCN2 cells after 24 h incubation in conditioned medium with and without of Bafilomycin A1. Figure6 shows representative examples of HEK293/HCN2 for both experimental conditions. We measured fluorescence intensity of siRNA Cy-3 (red fluorescence) over all of the cell area. Cells were picked at random. The data are summarized in Fig.6B. The average siRNA Cy-3 fluorescence intensity of HEK293/HCN2 cells was ∼16 fold higher (pink bar) than those incubated with Bafilomycin A1 (green bar). In comparison HEK293 cells directly transfected with 150 nmol/L of siRNA exhibited ∼20 and ∼3000 fold higher fluorescence intensity (gray bar), respectively (see Figure 6 legend for details). Figure6C shows summary histograms of siRNA Cy-3 fluorescence intensity distribution in the cells. The HEK293/HCN2 cells incubated with Bafilomycin A1 exhibited very low red fluorescence intensity over the narrow range (green histogram), whereas cells incubated in conditioning medium without Bafilomycin A1 yielded much higher fluorescence intensity in red spectra over a much broader intensity range (pink histogram). Experiments with HeLaCx43/HCN2 cells yielded similar results (not shown).

These data confirm that the extracellular pathway is a potential delivery pathway for siRNA that is inhibited by Bafilomycin A1.

### Tagged morpholinos targeting HCN2 can effectively reduce HCN2-induced currents

**Figure 7:**
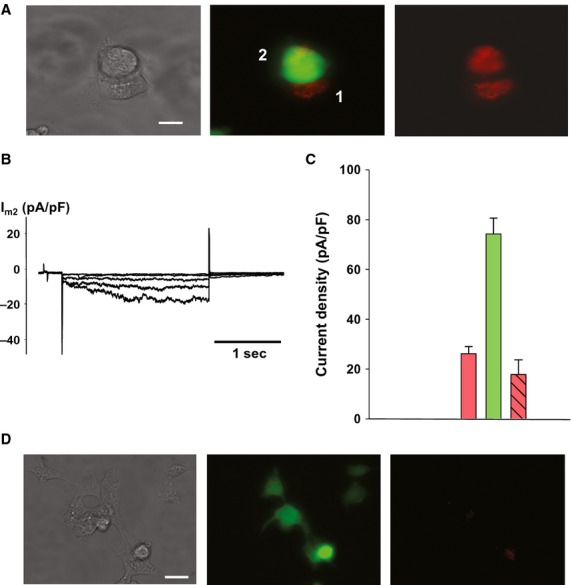
Morpholino transfer. (A) HEK293 cell pair where cell 2 is expressing eGFP and HCN2 and the cell 1 has been transfected with a red-tagged morpholino targeting HCN2. (B) Typical *I*_m_ recording from a recipient cell of a pair, indicating inhibited HCN2 currents (*g*_j_ = 14 nS). Scale bar, 10 μm. (C) Summary plot of HCN2 currents recorded from HEK293/HCN2 cells. Green bar: single control cells (74.3 ± 6.3 pA/pF, *n* = 43); pink bar: single cells transfected with morpholino (26.2 ± 2.8 pA/pF, *n* = 50); striped pink bar: recipient cells of a pair (17.8 ± 5.9 pA/pF, *n* = 5); *P* = 0.473. (D) HEK293/HCN2 cells cultured for 24 h in conditioned media obtained from morpholino-transfected HEK293 cells. No significant morpholino transfer via the extracellular space is observed. Scale bar, 20 μm.

Morpholinos are analogs to siRNAs that are also able to silence genes. We had a red-tagged morpholino made to target HCN2 and transfected it into HEK293 cells and then cocultured those cells with HEK293 cells expressing eGFP and HCN2 for 24 h. An example is shown in Fig.7A showing transfer from the source cells to the recipient eGFP-expressing cell. Figure7B illustrates an example of the HCN2-induced currents in a eGFP-expressing cell of a pair. Figure7C shows a summary of all the data in histogram form. The pink bar represents single eGFP cells directly transfected with morpholino while the green bar represents eGFP cells not transfected with morpholino. The striped bar is the average HCN2-induced current density in eGFP cells of cell pairs. To determine if there was an extracellularly mediated transfer a conditioned media experiment was performed and no significant amount of tagged morpholino could be found in the HEK293 cells (Fig.7D).

## Discussion

Here, we demonstrate that an intercellular delivery of siRNA exclusive of the extracellular space occurs alongside a separate extracellular-mediated pathway that is dependent on endosomal activity within cells. We make these observations by studying the magnitude of membrane current expressed following transfection with an exogeneous gene HCN2. Both siRNA and morpholinos targeting HCN2 are capable of using both the gap junction and extracellular pathways (although not with the same frequency). These observations represent the first report using biophysical measurements demonstrating multiple pathways for the delivery of silencing molecules. It is also the first report suggesting that the molecular makeup of the silencing molecule can help to determine the degree to which each of these two delivery pathways is used.

Previously, we demonstrated visually that morpholinos, an siRNA analog, are able to pass from cell to cell via gap junctions. We also demonstrated in the same study that siRNA's could generate a functional knockdown of an expressed gene (Valiunas et al. 2005) via cellular delivery. To illustrate gap junction-mediated transfer Valiunas et al. (2005) used a fluorescently tagged morpholino delivered by whole-cell patch clamp to one cell of a pair and monitored intensity in the source cell and the recipient cell. Junctions composed of connexin43 (Cx43) readily transfer morpholino within minutes. Figure1C of this study using a Cy-3-tagged siRNA shows a similar behavior. To demonstrate siRNA transfer-Valiunas et al. (2005) transfected NRK cells and MB16tsA fibroblasts with the vector, pH 1P-pgkneoB that directs the expression of a short hairpin RNA (shRNA) that inhibits the transcription of DNA polymerase *β* (pol *β*) a DNA repair enzyme (Polosina et al. 2004; Valiunas et al. 2005). The data demonstrated that transfer of siRNA targeting pol *β* is dependent upon the type of connexin expressed. A more recent study by Lim et al. (2011) provides evidence that exocytotic/endocytotic mechanisms are able to deliver miRNA/siRNA as well as gap junctions. Here, we provide evidence illustrating both pathways are effective in vitro. Bafilomycin A1 inhibition of extracellularly mediated delivery of siRNA reveals that gap junction-mediated siRNA transfer occurs and effectively reduces expression, as determined by monitoring HCN2-induced currents in target cells. The ability of siRNA targeting HCN2 to successfully reduce HCN2-induced currents in the presence of Bafilomycin A1 suggests that not all siRNAs or morpholinos traffic in the same manner within the intracellular compartments of a transfected cell. In this case the morpholino targeting HCN2 must be at a higher concentration within the cytoplasm of the source cell to be delivered to the target cell in the absence of extracellular delivery.

Bafilomycin A1 reduced extracellular delivery. But as the data of Fig.4 illustrate, gap junction-mediated delivery occurs in the presence of the drug. Since Bafilomycin A1 essentially retards extracellularly mediated traffic to and from the plasma membrane, no change in junctional conductance would be predicted. In fact, junctional conductance is reduced by Bafilomycin A1 22% in HeLa cells and 54% in HEK293 cells. If the drug was completely effective in blocking vesicular trafficking to and from the plasma membrane then one might assume junctional conductance would remain unchanged upon exposure to Bafilomycin A1. Our data does demonstrate a reduction in junctional conductance, but even in this circumstance where the signal to noise ratios might be reduced, cell pairs remained sufficiently coupled to result in effective delivery of siRNA targeting HCN2. The half-life of Cx43 has been reported to be between 2–5 h (Leithe and Rivedal 2007) thus the reduction we have observed suggests that Bafilomycin A1 is more effective in inhibiting trafficking to the plasma membrane than trafficking from it.

In vitro both gap junction and extracellularly mediated delivery are effective in reducing HCN2-induced currents in recipient target cells. The data illustrate that the extracellular (exocytotic/endocytotic) pathway, common to all cells, is a delivery pathway of potential use therapeutically. The most telling aspect of the exocytotic/endocytotic pathway for in vivo delivery is the dilution effect caused by the semi-infinite interstitial space. Even with a relatively small defined volume used in the conditioned media experiments the dilution effect is clear (∼20×). In vivo the extracellular volume (which can be near infinite) has the potential to dramatically reduce the effective concentration of deliverable siRNA. Gap junction-mediated delivery occurs in the presence of Bafilomycin A1 also indicating it to be a successful delivery pathway as well.

In all the experiments shown here delivery cells were directly transfected with siRNA or morpholino. We did not attempt to create a stably transfected cell line able to express an shRNA targeting HCN2 for two reasons: (1) stable transfection has proven difficult; and (2) the effective concentration in the transfected cell is unknown and most likely significantly lower than the amounts of siRNA/morpholino we can directly transfect (picomolar vs. nanomolar). A therapeutic advantage of allogenic cells loaded with a finite amount of siRNA/morpholino is a pulse-like delivery where the allogenic cell types will eventually be recognized by the immune system and destroyed.

Ultimately the therapeutic success of cellular delivery will be defined in vivo. The relative contributions of the two pathways for delivery are yet to be determined for these in vivo clinical conditions.
